# Healthcare judicialization: an analysis of indicators and official data on medications

**DOI:** 10.1590/0034-7167-2022-0413

**Published:** 2024-07-29

**Authors:** Nádia Bernardinis, Vanessa Terezinha Gubert, Cristiane Munaretto Ferreira, Jorge Otávio Maia Barreto

**Affiliations:** I Universidade Federal de Mato Grosso do Sul. Campo Grande, Mato Grosso do Sul, Brazil; II Fundação Oswaldo Cruz. Brasília, Distrito Federal, Brazil

**Keywords:** Health Litigation, Pharmaceutical Services, Health Services Accessibility, Right to Health, Process Assessment, Health Care, Judicialización de la Salud, Servicios Farmacéuticos, Accesibilidad a los Servicios de Salud, Acceso a Medicamentos Esenciales y Tecnologías Sanitarias, Indicadores de los Resultados

## Abstract

**Objectives:**

to analyze judicial demands for medications in Campo Grande, Brazil, between July 2018 and June 2020.

**Methods:**

the four dimensions of the Manual of Indicators for Evaluation and Monitoring of Judicial Demands for Medications were examined.

**Results:**

676 judicial processes were identified, corresponding to 1006 requests for 284 different medications. In 92.74% of the processes, access to medications was granted, with 88.80% granted on an urgent basis. The median time between the decision and delivery of the medication was 146 days. The average monthly cost of acquiring medications was R$ 2,183.68 Brazilian reais. Among the identified medications, 90.22% had at least one therapeutic alternative available in the public healthcare system.

**Conclusions:**

characterizing and analyzing judicial demands related to medications can support discussions on updating medication lists and clinical protocols, organizing healthcare services, allocating resources, and implementing actions to reduce judicialization.

## RESUMEN

**Objetivos:**

analizar las demandas judiciales de medicamentos en Campo Grande, Brasil, entre julio de 2018 y junio de 2020.

**Métodos:**

se analizaron las 4 dimensiones del Manual de Indicadores de Evaluación y Monitoreo de Demandas Judiciales de Medicamentos.

**Resultados:**

se identificaron 676 casos judiciales, correspondientes a 1006 solicitudes para 284 medicamentos diferentes. En el 92,74% de los casos, se concedió acceso a los medicamentos, siendo el 88,80% de ellos de carácter urgente. El tiempo mediano entre la decisión y la entrega del medicamento fue de 146 días. El costo medio mensual de adquisición de los medicamentos fue de R$ 2.183,68 reales. Entre los medicamentos identificados, el 90,22% tenían al menos una alternativa terapéutica disponible en el sistema público de salud.

**Conclusiones:**

la caracterización y análisis de las demandas judiciales relacionadas con medicamentos pueden respaldar las discusiones sobre la actualización de las listas de medicamentos y los protocolos clínicos, la organización de los servicios de salud, la asignación de recursos y las acciones para reducir la judicialización.

## INTRODUCTION

Healthcare expenditure constitutes a significant portion of Brazil’s Gross Domestic Product (GDP), reaching approximately 10%, with consistent growth in recent years, both in service volume and costs^(1)^. The Unified Health System (SUS in Portuguese) is constitutionally committed to ensuring universal, comprehensive, and equitable health coverage^(2)^, including free access to medications, especially those listed in the National List of Essential Medicines (RENAME). However, since its inception, SUS has been underfunded, often leading to rationing of pharmaceutical services and limitations and intermittencies in medication stocks^(3)^.

Resource scarcity and the diversity of epidemiological patterns, coupled with low standardization of clinical conduct, make it difficult to determine the health needs of different populations, impacting health management priorities. The so-called “judicialization of healthcare” is an expression of this competition for resources, which has been reaching increasingly significant levels in national socioeconomic relations^(1)^. A large portion of these judicial demands aims at accessing pharmaceutical products not incorporated into SUS, despite the availability of therapeutic alternatives. This is due to the current healthcare model, in which physicians are unaware of the items standardized in SUS and do not consider evidence in their prescriptions, seeking what is new in the market^(4)^.

The number of healthcare-related judicial demands increased by 130% in the country between 2008 and 2017^(1)^. In the Mato Grosso do Sul State Court of Justice (TJMS), there was an exponential increase in first-instance cases, rising from 39 in 2008 to 5,825 in 2017, and in second-instance cases, from 13 in 2008 to 2,950 in 2017^(1)^. In this context, the exacerbation of judicial processes with the interim granting of demands is evident, aiming solely at medication access, as reservations were rare^(5-8)^.

The Manual of Indicators for Evaluation and Monitoring of Judicial Demands for Medications^(6)^ presents a series of indicators aimed not only at identifying difficulties but also at creating opportunities for well-informed action by healthcare managers and the judicial system. This aims to develop strategies, instruments, and mechanisms to improve pharmaceutical care and reduce the intensity of judicial actions^(9-10)^. On the other hand, litigation in healthcare can also generate a positive social impact, motivating the creation of policies that promote better access to healthcare and even structural changes, improving the distribution of social goods and public services among society^(11-12)^.

The four dimensions of analysis in the Manual address the sociodemographic characteristics of the authors of the actions, procedural, medical-health, and political-administrative aspects. These categories are subdivided into indicators that allow for characterizing the socioeconomic situation of the plaintiffs, planning agile service flows, and identifying possible deficiencies in Pharmaceutical Assistance management. This information can support discussions on the implementation or updating of Clinical Protocols and Therapeutic Guidelines, as well as changes in the disease profile, and help identify the efficacy, safety, favorable costs, and risk/benefit of prescribed medications or the lack thereof^(6)^. Given the scarcity of evidence analyzing the phenomenon of medication judicialization within the scope of municipal public health, it was considered necessary to conduct and share this analysis.

## OBJECTIVES

To analyze judicial demands for medications in Campo Grande, Brazil, between July 2018 and June 2020.

## METHODS

### Ethical aspects

The study was authorized by the Municipal Health Department of Campo Grande - MS and approved by the Research Ethics Committee of the Dom Bosco Catholic University.

### Study design, period, and location

This is an observational, cross-sectional study conducted in the Judicial Procurement Division and the Division of Dispensation of Judicial Supplies (DDIJ) of the Procurement and Bidding Management Division of the Municipal Health Department of Campo Grande, from July 2018 to June 2020. The current text used the STROBE tool as a guide for writing the study report, meeting the criteria of the tool related to title, abstract, introduction, methods, results, and discussion.

### Population or sample and inclusion and exclusion criteria

The unit of analysis was the individual judicial process, including processes requesting medications and filed by citizens against the municipality of Campo Grande - MS. Processes under judicial secrecy, according to the Civil Procedure Code, were excluded.

The obtained information was systematized in an Excel® spreadsheet and analyzed according to the indicators described in the Manual of Indicators for Evaluation and Monitoring of Judicial Demands for Medications^(6)^ (Chart 1).

**Chart 1:** Indicators Analyzed in the Four Dimensions of Judicial Demands in Campo Grande, Mato Grosso do Sul, Brazil

DIMENSION 1	INDICATOR
Sociodemographic characteristics of the plaintiff - population characteristics regarding social and demographic aspects	1 - Proportion of the population by age group 2 - Proportion of the population by occupation 3 - Proportion of the population by municipality of residence of the plaintiff 4 - Monthly family income
**DIMENSION 2**	**INDICATOR**
Procedural characteristics of judicial actions - aspects that comply with national and local laws	1 - Median time for interim decision or preliminary injunction in the first instance 2 - Median time for medication delivery 3 - Proportion of interim decisions or preliminary injunctions granted 4 - Proportion of judgments favorable to the plaintiff 5 - Ratio of collective judicial actions 6 - Proportion of judicial actions filed by type of defendant
**DIMENSION 3**	**INDICATOR**
Medical and health characteristics of judicial actions - aspects related to the body of knowledge of Health Sciences	1 - Proportion of medications by therapeutic/pharmacological/chemical substance subgroups 2 - Proportion of medications prescribed by generic name 3 - Proportion of requested medications listed in current essential medication lists 4 - Proportion of judicial actions containing additional documents, other than medication prescriptions 5 - Proportion of medications with recommendation strength Classes I and IIa in therapeutic indication 6 - Proportion of main diagnoses by diagnostic category 7 - Proportion of patients registered in the healthcare instance prior to judicial demand 8 - Medication demand expenditure ratio 9 - Proportion of demanded medications with therapeutic alternatives in the SUS
**DIMENSION 4**	**INDICATOR**
Political-administrative characteristics of judicial actions - aspects related to the executive, administrative, and economic competencies of the Public Administration. In this case, it refers to the management of Pharmaceutical Assistance in the Unified Health System	1 - Proportion of medications registered with ANVISA (National Health Surveillance Agency) 2 - Proportion of medications by component of the Pharmaceutical Assistance funding block 3 - Proportion of judicial actions that include at least one medication prescribed for “off-label” use 4 - Proportion of judicial actions demanding at least one medication that is outside the components of the Pharmaceutical Assistance funding block 5 - Proportion of judicial actions demanding at least one medication from the Specialized Component of Pharmaceutical Assistance

### Study protocol

This protocol was made available on the free OSF platform (https://osf.io/32vyg). The indicators were synthesized and described according to absolute values, proportions, and ratios. As provided, they were classified into four dimensions related to the characteristics: 1) sociodemographic of the plaintiff; 2) political-administrative; 3) procedural of judicial actions; and 4) medical-health characteristics of actions.

### Results analysis and statistics

Data were collected from information available at https://esaj.tjms.jus.br/ and from DDIJ files, concerning processes meeting the inclusion criteria.

In dimension 1, which analyzes the sociodemographic characteristics of the plaintiff, data such as age in years, occupation, municipality of residence of the plaintiff, and family income in minimum wages were collected. For calculating minimum wages, the value in force in each analyzed year was considered. Proportions were calculated as the ratio of the number of patients to the total population, multiplied by 100.

In dimension 2, regarding the procedural characteristics of judicial actions, data related to the median time for interim decision or preliminary injunction in the first instance and for medication delivery, in days, were presented. The median value or the simple arithmetic mean between the two median values of the distribution in ascending order was calculated.

The proportions of interim decisions or preliminary injunctions granted, judgments favorable to the plaintiff, and judicial actions filed by type of defendant were calculated as the ratio of the number of actions related to the indicator to the total number of judicial actions, multiplied by 100.

In dimension 3, related to medical-health characteristics, medications were classified according to the recommendation of the World Health Organization, using the Anatomical Therapeutic Chemical (ATC) code^(13)^ for specification regarding therapeutic/pharmacological/chemical substance subgroups. The proportion was calculated as the ratio of the number of medications in the subgroup to the total number of medications demanded, multiplied by 100.

For the indicator “proportion of requested medications listed in current essential medication lists,” the RENAME 2020^(14)^, the State List of Essential Medicines (RESME) 2020^(15)^, and the Municipal List of Essential Medicines (REMUME) 2016^(16)^ were used. For the proportion of main diagnoses by diagnostic category, the classification was made according to the tenth revision of the International Classification of Diseases (ICD-10).

The medications prescribed by generic name, the judicial actions containing additional documents beyond medication prescriptions, and the number of patients registered in the healthcare system prior to judicial demand were verified in the judicial records and expressed as percentages. The proportion of medications with recommendation strengths Classes I and II in therapeutic indication, and those with therapeutic alternatives in the SUS, related to medications that could technically be interchangeable with other standardized medications due to therapeutic equivalence, were expressed as percentages. The medication expenditure ratio was calculated based on the commitments issued for acquisition. For the indicators of dimension 4, which analyze the political-administrative characteristics of judicial actions, medications registered with the National Health Surveillance Agency (ANVISA in portuguese) and with off-label use indications were evaluated. In the analysis of medications by component of the Pharmaceutical Assistance funding block, the RENAME 2020^(14)^ was used to verify items not belonging to and belonging to the Specialized Component. The calculations related to this dimension were expressed as a percentage.

## RESULTS

The DDIJ received 676 processes with a judicial determination for the provision of medications from July 2018 to June 2020. When analyzing the indicators proposed in the Manual of Indicators for Evaluation and Monitoring of Judicial Demands for Medications, it was observed that 87 (12.87%) processes contained all the data regarding the listed indicators for analysis, while 589 (87.13%) had incomplete data.

The processes, in general, contained one or more medication requests. Thus, all 676 processes were analyzed, resulting in 1006 medication requests, with an average of 1.49 medications requested per process. The requests involved 284 medications with different active ingredients.

### Dimension 1 - socio-demographic characteristics of the plaintiff

The majority of plaintiffs in the judicial action were aged between 70 and 79 years (14.20%), with an average age of 48.85 years, ranging from three months to 94 years. Nearly half were retired or pensioners (43.46%), and 10.87% were homemakers. Table 1 accounts for 86.89% of the plaintiffs’ occupations, including only occupations with a percentage greater than or equal to 5.00%.

**Table 1:** Socio-demographic characteristics of the plaintiff in the judicial action (N=676)

	n	%
Age range (years)		
Less than 1 year	4	0.59
1 to 9 years	42	6.21
10 to 19 years	43	6.36
20 to 59 years	295	43.63
60 to 79 years	186	27.51
80 years or more	49	7.25
No information	57	8.43
Occupation		
Retired or pensioner	236	43.46
Homemaker	59	10.87
Unemployed	41	7.55
Student	29	5.34
No information	133	19.67

Of the plaintiffs, 93.05% resided in Campo Grande. Absence of information regarding age range, occupation, and domicile of the plaintiffs was observed in 57 (8.43%), 133 (19.67%), and 47 (6.95%) cases, respectively.

The average monthly family income of the plaintiffs was 1.47 ± 0.94 minimum wages, with a range from 0.17 to 9.77 minimum wages, considering the value of the minimum wage for each year analyzed. Lack of information regarding income was observed in 266 (39.35%) cases, as shown in Table 2. There was no significant difference when comparing the income means between the analyzed semesters.

**Table 2:** Distribution of Monthly Family Income of Lawsuit Plaintiffs per Year in Campo Grande, Mato Grosso do Sul, Brazil (2018-2020)

Income	2018	2019	2020	Total	
	n	n	n	n	%
Up to 0.5 minimum wage	1	3	0	4	0.59
>0.5 to 1 minimum wage	19	37	16	72	10.65
>1 to 3 minimum wages	112	146	43	301	44.53
>3 to 5 minimum wages	11	12	6	29	4.29
>5 to 11 minimum wages	1	1	2	4	0.59
No information	101	127	38	266	39.35
Total	245	326	105	676	100

*Minimum Wage: 2018 - R$ 954.00^(17)^; 2019 - R$ 998.00^(18)^; 2020 - R$ 1,039.00^(19)^.*

### Dimension 2 - procedural characteristics of judicial actions

All actions had a single plaintiff, and the defendant was the Municipal Health Department of Campo Grande-MS, with no collective actions or joinder observed in the active role during the study period. Interim relief was granted in 600 cases (88.76%), while in 76 (11.24%) cases, there was no record of this occurrence. The judge granted provisional relief - right at the beginning of the action - by supplying the requested medications in 1,005 (99.90%) requests. In one case, access to one of the requested medications was denied.

The median time between the start of the process and the decision granting access was 34 days for the 616 processes that contained the information. In the remaining 60 processes, there was no information regarding the start date of the judicial action. There was no significant difference when comparing the time averages between the request and the decision between the semesters.

The delivery of requested medications occurred in 419 (41.65%) requests, with a median time between the decision and effective delivery of 146 days. The justifications for not delivering the remaining 587 (58.35%) requests were: i) medication acquisition issues in 380 requests (37.77%), ii) treatment suspension by the physician in 86 (8.55%), iii) death in 71 (7.06%), and iv) lack of contact with the patient in 50 (4.97%).

### Dimension 3 - medical-health characteristics of judicial actions

In the 1006 requests, 284 medications with different active ingredients were requested. Of these, 255 were classified up to the fifth level of the ATC Classification. There was a predominance of medications acting on the Nervous System (22.54%), Antineoplastic and Immunomodulating Agents (16.20%), Digestive System and Metabolism (14.08%), and Cardiovascular System (12.68%). Twenty medications (7.04%) did not have classification, of which three (1.05%) were classified up to the fourth level and five (1.76%) up to the third.

In 547 (54.37%) of the 1006 requests, medications were prescribed by generic name, 911 (90.56%) had therapeutic indication for the mentioned diagnosis in the records, and 944 (93.80%) had an alternative for treatment in the SUS.

Additionally, in 567 (83.88%) of the 676 actions, it was possible to identify additional documents, such as specific exams and medical reports, to support the diagnosis and request. No information was identified regarding patient registration in a health instance prior to the judicial process. Technical Support Nucleus (NAT Jus) opinions were present in 868 (86.28%) of the requests, with the majority being unfavorable to medication provision, 532 (52.88%), and 336 (33.40%) being favorable. The opinion was not present in 138 (13.72%) of the medication requests. The proportion of patients from SUS treatment processes was 385 (56.95%), 220 (32.54%) from other services, and 71 (10.50%) without this data.

Table 3 presents data on the presence in the cited official lists for the 284 medications requested in the processes. Ninety-nine (34.86%) medications are present in RENAME, with hydralazine, lactulose, eculizumab, and hydrocortisone acetate only present on this list. Bromopride, diosmin with hesperidin, cilostazol, and fenoterol are only covered in REMUME. Dapagliflozin and sacubitril with valsartan are only present in RESME. There are medications that belong simultaneously to all lists, such as insulin glargine and aspart and ciprofibrate.

**Table 3:** Inclusion of Medications Requested Judicially in Official Medication Lists in Campo Grande, Mato Grosso do Sul, Brazil (2018-2020)

Official Lists	n	%
RENAME	99	34.86
REMUME	57	20.07
RESME	41	14.43
RENAME, REMUME AND RESME	3	1.06
Does not belong	183	64.44
Total	284	100

*RENAME - National List of Essential Medicines; REMUME - Municipal List of Essential Medicines; RESME - State List of Essential Medicines.*

The sum of the three official medication standardization lists does not total 100% because there are medications that belong to only one list.

The most requested medications were enoxaparin 40mg (86; 8.55%), tiotropium bromide 2.5mcg (69; 6.86%), rivaroxaban 20mg (64; 6.36%), and insulin glargine and glulisine (74; 7.35%).

In 626 (92.60%) cases, it was possible to identify at least one diagnosis, totaling 244 morbidities. There were cases mentioning several ICD codes. The lack of this information was observed in 50 (8.40%) cases. The most frequent morbidities are shown in Table 4.

**Table 4:** Distribution of Main Diagnoses in Judicial Processes According to the International Classification of Diseases (ICD-10) in Campo Grande, Mato Grosso do Sul, Brazil (2018-2020)

ICD	Second semester 2018	First semester 2019	Second semester 2019	First semester 2020	Total	%
C61 - Malignant Neoplasm of Prostate	8	2	4	3	17	1.69
D68 - Coagulation Defects	23	26	14	9	72	7.16
E10 - Insulin-dependent Diabetes Mellitus	28	19	22	11	80	7.95
F33 - Recurrent Depressive Disorder	7	1	2	5	15	1.49
I10 - Essential (Primary) Hypertension	13	7	5	6	31	3.08
J44 - Other Chronic Obstructive Pulmonary Diseases	19	8	34	5	66	6.56
J45 - Asthma	7	5	1	3	16	1.59
M79 - Other Soft Tissue Disorders. Not Elsewhere Classified	10	-	2	3	15	1.49

The average cost of medications provided for the 419 requests, according to the commitments issued, was R$ 399.45. Ocrelizumab and alemtuzumab were the medications with the highest dose cost, at R$ 25,285.98 and R$ 24,882.05, respectively. Gliclazide has the cheapest tablet, costing R$ 0.04. Considering monthly treatment, the average cost was R$ 2,183.68.

The transfer of federal financial resources is R$ 5.90 per inhabitant/year, in addition to the state and municipal counterparties, which must be at least R$ 2.36 per inhabitant/year^(14)^. Thus, the annual investment for the acquisition of medications per citizen of Campo Grande is at least R$ 10.62. The cost for granting monthly treatment through the judiciary was 2,467 times higher than this minimum value.

### Dimension 4 - political-administrative characteristics of judicial actions

The distribution of medications according to the component of the Pharmaceutical Assistance funding block is detailed in Table 5. There is a predominance (64.79%) of requests for medications that are not included in the components. According to RENAME, 100 medications (35.21%) are part of the funding block components, and some medications are included in more than one component simultaneously.

**Table 5:** Classification of Medications Judicially Requested by SUS Financing Block in Campo Grande, Mato Grosso do Sul, Brazil (2018-2020)

Medication by funding block	n	%
Basic	52	18.31
Strategic	6	2.11
Specialized	41	14.44
Basic and Specialized	1	0.35
Outside funding components	184	64.79
Total	284	100

The judicial actions that demanded at least one medication not included in the funding block components totaled 443 (65.53%), and those that requested at least one medication from the Specialized Component amounted to 245 (36.24%).

All 284 medications are registered with ANVISA, and three of them had off-label indications. Rituximab, pembrolizumab, and cannabidiol do not have indications in the label for systemic lupus erythematosus, pemphigus foliaceus, severe pemphigus vulgaris, malignant neoplasm of the eye and adnexa, and Parkinson’s disease, as mentioned in the records.

## DISCUSSION

Despite the Manual of Indicators for Evaluation and Monitoring of Judicial Demands for Medications assisting health and judicial system managers in identifying difficulties, developing strategies, and improving tools or mechanisms to enhance pharmaceutical assistance and reduce the volume of judicial actions, local publications that utilize these indicators are scarce, as are national assessments of the quality of action and judicial decisions^(9)^.

Furthermore, the unavailability of data, both complete and partial, was found in 87.13% of cases. The lack of traceability after the judicial decision and the failure to analyze the data present in the cases hamper the assessment of impact, costs, investment, the possibility of standardizing new medications, and the improvement of pharmaceutical assistance. Traceability after the judicial decision would include information on medication receipt, patient use, and treatment efficacy. Regarding data analysis, it is important to note that it was conducted in this research as a prerequisite for obtaining a degree in stricto sensu postgraduate (master’s) studies, but in routine conditions, such analysis is not a judicial service practice. Thus, the absence of routines for data collection, processing, and analysis complicates decision-making by public entities to reduce the impact of judicialization on their budgets.

In the analysis of procedural characteristics of judicial actions, most requested medications were granted. Only one request was denied because there was a standardized alternative in RENAME, and there was no evidence, through a medical report, of the indispensability of the prescribed medication or the ineffectiveness of the drug provided by the SUS. This decision against providing the prescribed medication, based on multidisciplinary analysis as stated in the case file opinion, and verification with official lists for the presence of a standardized alternative in SUS, enables effective actions and promotes optimization of resources applied^(20-21)^.

The slowness and low percentage of medication supply compromise treatments, especially for patients in advanced disease stages and at risk of death. The requirements and deadlines inherent in the medication acquisition process may justify this delay in meeting the demand. Therefore, awareness of this bureaucracy, as well as the time for medication supply, should be considered by the judiciary in issuing the verdict, as well as by the physician and the patient to avoid treatment compromise.

In the findings of dimension three, the prescription of medication by its generic name, originating from the SUS, was observed in the majority of legal actions, as well as in actions in Rio de Janeiro^(22)^ and the Federal District^(23)^. Conversely, the opposite was evidenced in actions from the states of Rio Grande do Norte^(24)^ and Minas Gerais^(5)^, where prescriptions mainly originated from private healthcare services. This predominance of prescriptions originating from SUS suggests unawareness or non-adherence of prescribing professionals to official lists and clinical protocols. The lack of continued education, with dissemination and support regarding clinical guidelines and standardized medications, may lead to increased judicialization. Irregular access to medications provided by SUS is another aggravating factor.

In most legal proceedings, there was the presence of additional documents. These documents can guide the updating and incorporation of new medications into official lists and the implementation of clinical protocols since they may contain information about the ineffectiveness of the drug provided by SUS and the proposed new medical approach.

The presence of opinions from the Technical Support Nucleus (NAT Jus), mostly unfavorable due to the existence of a standardized alternative in SUS or the lack of evidence of the indispensability of the prescribed medication, shows that recommendations are not always followed, given that the majority of medications were granted. However, providing the requested treatment does not guarantee the intended effectiveness and can intensify judicialization. Evidence-informed judicial decisions and the use of clinical protocols as a technical parameter contribute to providing safe and effective medications for the population^(6,25)^.

A large portion of the requested medications is not present in the cited official lists. The same was found in studies in the states of São Paulo^(4)^, Minas Gerais^(5)^, and Rio Grande do Norte^(24)^. However, there are medications present only in RENAME that, by being part of the national list, have financial resources allocated for their acquisition and could be incorporated into the local medication list.

The adoption of specific and complementary medication relationships is legally supported^(26)^ and should address the main health problems and local programs. The inclusion of medications exclusively in REMUME and RESME, as observed in this research, optimizes the resources invested in medication acquisition and increases access since purchases can cater to a larger number of patients, increasing the quantity acquired and, consequently, competition in bidding processes.

The shortcomings in medication acquisition logistics, the prescribers’ unawareness of official lists, and the requirements for obtaining treatment also serve as justifications for resorting to judicial means. This can be exemplified by the most requested medications such as enoxaparin and the insulins glargine and glulisine, which are included in RESME and have acquisition requirements outlined in the Clinical Protocols and Therapeutic Guidelines (PCDT).

Chronic conditions like diabetes mellitus, chronic obstructive pulmonary diseases, and hypertensive diseases were the most frequent diagnoses, as also evidenced in São Paulo^(4)^ and Rio Grande do Norte^(24)^. The predominance of elderly patients found in the research, along with the aging of the general population, may account for these frequent diagnoses, the need for continuous treatments^(4)^ - often high-cost - and the resort to judicial requests.

Furthermore, the results indicate judicialization to access medications related to treatments of chronic conditions that require primary care and could have been addressed in Primary Health Care.

According to the commitments made for medication acquisition, the cost for granting monthly treatment through judicial means was 2,467 times higher than the investment for medication acquisition. This allocation of resources contradicts the principle of equity, potentially leading to access inequalities, as it prioritizes individual needs and disregards the collective. The Pharmaceutical Assistance policy seems not to be observed, which may jeopardize the planning and administration of resources, which are scarce and should be outlined by health policies.

In the political-administrative characteristics of the analyzed judicial actions, off-label use of three medications was observed, considering the diagnoses mentioned in the records and the label. Off-label use of a medication may imply that it does not produce the expected effect, is ineffective, or unsafe. Granting it for this purpose may require a comprehensive multiprofessional evaluation to verify if the request is technically and therapeutically justifiable, in addition to pharmacovigilance monitoring to identify, assess, and monitor adverse events and ensure that benefits outweigh risks^(27)^.

Ensuring adequate patient monitoring, together with granting medication supply through judicial means, contributes to ensuring effective access to health and justice, promoting rational medication use, and avoiding potential fraud or misapplication of public resources. Monitoring health outcomes, obtaining data regarding the necessity of maintaining the benefit, and mediating the relationship between the demanding user and the judicial system can lead to around a 30% reduction in overall costs^(28-29)^.

Even as it addresses individual needs, judicialization can still have positive effects by stimulating discussions about updating programs and protocols. This provides an opportunity for analysis to integrate new medications into official lists or correct flaws related to medication acquisition.

Despite strategies existing to improve the Brazilian public administration’s management of healthcare judicialization, these are not mandatory and lack guarantees of implementation by public institutions^(30)^. We emphasize the potential use of the Evaluation and Monitoring Indicators of Medication Judicial Demands as a tool to foster closer collaboration between managers and the judiciary for process analysis and improvement of health-related policies, promoting comprehensive access to healthcare, and enhancing the distribution of social goods and public services among society^(11-12,31)^.

Despite a gap in specific technical knowledge among judges, requesting evidence-based information and using protocols as a benchmark can provide judges with greater confidence, as there will be an explicit objective technical reference capable of impacting the reformulation of public health policy^(32)^.

Widespread dissemination of data from assessments of judicial demands, official medication lists, and the development and implementation of guides and manuals for clinical practice guidance could optimize resource allocation, contribute to expanding access to healthcare, and potentially reduce the intensity of judicialization.

Integration of multidisciplinary healthcare and legal teams, with requests for evidence-based information, along with communication about the continuation or suspension of requested treatment through judicial demand, brings effectiveness to both legal and healthcare access.

### Study limitations

The lack of access to medical prescriptions or their copies and other medical documents appended to the judicial process were significant limitations of this research. It is worth noting that the Municipal Health Department does not have a systematization of judicial processes, and the construction of the database was manually carried out through digitized copies of the judicial processes, which may accentuate the gaps highlighted in the research.

### Contributions to the nursing, health, and public policy areas

The study significantly contributes to the nursing field by demonstrating that with knowledge of the peculiarities of judicial demands, it is possible to better structure compliance. The provision of medication, the main objective of the demand, can be accompanied by continuous support to the user by the entire healthcare team, promoting rational and safe medication use. The implementation of practical protocols and the training of prescribing professionals, sensitizing them to the treatments available in the SUS, also contribute to the organization of health services and medication-related policies.

For the health and public policy areas, the evaluation of medications and protocols for frequent updates, as well as the systematization of the entire work process, by creating routines for analyzing data from judicial demands and promoting dialogue between managers and the judiciary, contribute to optimizing the resources employed.

## CONCLUSIONS

The analysis of judicial demands in the municipality of Campo Grande, through the listed indicators, provides subsidies for comparisons with other locations and for the dimensioning of judicialization in the municipality. Despite the limitations of the study, with knowledge of the peculiarities of the demands, there is collaboration for the direction of medication-related policies and the reduction of judicialization.

However, new evaluative or benchmarking research that assesses indicators related to judicial demands for medication access is useful for improving access to medication and optimizing the resources employed for its compliance, leading healthcare teams to promote rational and safe medication use.
