# Supplementary material for: Healthcare judicialization: an analysis of indicators and official data on medications
Source: Rev Bras Enferm. 2024 Jul 29;77(3):e20220413. doi: 10.1590/0034-7167-2022-0413 (PMC11290738; doi:10.1590/0034-7167-2022-0413)
Supplement: Translation

# Translation: Judicialização da saúde: uma análise de indicadores e dados oficiais sobre medicamentos

**DOI:** 10.1590/0034-7167-2022-0413pt

**Authors:** Nádia Bernardinis, Vanessa Terezinha Gubert, Cristiane Munaretto Ferreira, Jorge Otávio Maia Barreto

**Affiliations:** I Universidade Federal de Mato Grosso do Sul. Campo Grande, Mato Grosso do Sul, Brasil; II Fundação Oswaldo Cruz. Brasília, Distrito Federal, Brasil

**Keywords:** Judicialização da Saúde, Assistência Farmacêutica, Acesso aos Serviços de Saúde, Acesso a Tecnologias em Saúde, Indicadores

## Abstract

**Objetivos:**

analisar as demandas judiciais por medicamentos em Campo Grande, Brasil, entre julho de 2018 e junho de 2020.

**Métodos:**

foram analisadas as 4 dimensões do Manual de Indicadores de Avaliação e Monitoramento de Demandas Judiciais de Medicamentos.

**Resultados:**

676 processos judiciais foram identificados, correspondendo a 1006 solicitações para 284 diferentes medicamentos. Em 92,74% dos processos o acesso aos medicamentos foi concedido, sendo 88,80% em caráter de urgência. O tempo mediano entre a decisão e entrega do medicamento foi 146 dias. O custo médio mensal de aquisição dos medicamentos foi R$ 2.183,68 reais. Dentre os medicamentos identificados, 90,22% tinham pelo menos uma alternativa terapêutica disponível no sistema público de saúde.

**Conclusões:**

a caracterização e análise das demandas judiciais relacionadas a medicamentos pode apoiar as discussões sobre atualização das listas de medicamentos e protocolos clínicos, a organização dos serviços de saúde, a alocação de recursos e as ações para reduzir a judicialização.

## INTRODUÇÃO

A assistência à saúde é responsável por gastos expressivos, atingindo aproximadamente 10% do produto interno bruto (PIB) brasileiro, com consistente crescimento nos últimos anos, tanto em volume de serviços quanto em seus custos^(1)^. O Sistema Único de Saúde (SUS) está constitucionalmente comprometido em garantir cobertura universal, abrangente e igualitária às ações de saúde^(2)^, incluindo acesso gratuito a medicamentos, notadamente aos listados na Relação Nacional de Medicamentos Essenciais (RENAME). Porém, desde sua criação, o SUS é subfinanciado, levando muitas vezes ao racionamento dos serviços farmacêuticos e à limitação e intermitência dos estoques de medicamentos^(3)^.

A escassez de recursos e a diversidade dos padrões epidemiológicos, aliadas à baixa padronização da conduta clínica, dificultam a determinação das necessidades de saúde das diferentes populações, repercutindo sobre as prioridades da gestão de saúde. A chamada “judicialização da saúde” é uma expressão desta concorrência por recursos que vem atingindo níveis cada vez mais expressivos nas relações socioeconômicas nacionais^(1)^. Grande parte destas demandas judiciais visa ao acesso a produtos farmacêuticos não incorporados ao SUS, mesmo havendo alternativas terapêuticas disponíveis. Isso ocorre em virtude do modelo assistencial atual, no qual o médico desconhece os itens padronizados no SUS e não considera as evidências em suas prescrições, buscando o que é novidade no mercado^(4)^.

O número de demandas judiciais relativas à saúde aumentou 130% no país, entre 2008 e 2017^(1)^. No Tribunal de Justiça de Mato Grosso do Sul (TJMS), houve um aumento exponencial de processos na primeira instância, passando de 39 em 2008 para 5825 em 2017 e, na segunda instância, de 13 processos em 2008 para 2950 processos em 2017^(1)^. Neste contexto, percebe-se a exacerbação dos processos judiciais com o deferimento cautelar das demandas, tendo como objetivo apenas o acesso a medicamentos, uma vez que raras eram as ressalvas^(5-8)^.

O Manual de Indicadores de Avaliação e Monitoramento das Demandas Judiciais de Medicamentos^(6)^ apresenta uma série de indicadores que visam não só identificar dificuldades, mas também criar oportunidades para a ação bem informada de gestores da saúde e do sistema judicial, no sentido de desenvolverem estratégias, instrumentos e mecanismos para melhorar a assistência farmacêutica e reduzir a intensidade das ações judiciais^(9-10)^. Por outro lado, o litígio na área da saúde também pode gerar um impacto social positivo, motivando a criação de políticas que promovam melhor acesso aos cuidados de saúde e até mesmo a mudanças estruturais, melhorando a distribuição de bens sociais e serviços públicos entre a sociedade^(11-12)^.

As quatro dimensões de análise do Manual abordam as características sociodemográficas dos autores das ações, processuais, médico-sanitárias e político-administrativas. Estas categorias são subdivididas em indicadores que permitem caracterizar a situação socioeconômica dos demandantes, planejar fluxos de atendimentos ágeis e identificar possíveis deficiências na gestão da Assistência Farmacêutica. Essas informações podem apoiar as discussões sobre a implementação ou atualização de Protocolos Clínicos e Diretrizes Terapêuticas, bem como sobre mudanças no perfil de doenças, além de ajudar a identificar a eficácia, segurança, custos favoráveis, e o risco/benefício dos medicamentos prescritos ou a falta destas condições^(6)^. Diante da escassez de evidências que analisem o fenômeno da judicialização de medicamentos no âmbito da saúde pública municipal, considerou-se necessária a condução e o compartilhamento desta análise.

## OBJETIVOS

Analisar as demandas judiciais por medicamentos em Campo Grande, Brasil, entre julho de 2018 e junho de 2020.

## MÉTODOS

### Aspectos éticos

O estudo foi autorizado pela Secretaria Municipal de Saúde de Campo Grande - MS e aprovado pelo Comitê de Ética em Pesquisa da Universidade Católica Dom Bosco.

### Desenho, período e local do estudo

Trata-se de um estudo observacional, transversal, desenvolvido na Divisão de Compras Judiciais e na Divisão de Dispensação de Insumos Judicializados (DDIJ) da Gerência de Gestão de Compras e Licitações da Secretaria Municipal de Saúde de Campo Grande, no período de julho de 2018 a junho de 2020. O texto atual utilizou a ferramenta *STROBE* como guia para a escrita do relato do estudo, atendendo aos critérios da ferramenta relacionados ao título, resumo, introdução, métodos, resultados e discussão.

### População ou amostra e critérios de inclusão e exclusão

A unidade de análise foi o processo judicial individual, incluindo-se os processos que solicitaram medicamentos e foram movidos pelo cidadão contra o município de Campo Grande - MS. Foram excluídos os processos que tramitam em segredo de justiça, de acordo com o Código de Processo Civil.

As informações obtidas foram sistematizadas em uma planilha *Excel* ^®^ e analisadas segundo os indicadores descritos no Manual de Indicadores de Avaliação e Monitoramento das Demandas Judiciais de Medicamentos^(6)^ (Quadro 1).

**Quadro 1:** Indicadores Analisados nas Quatro Dimensões das Demandas Judiciais em Campo Grande, Mato Grosso do Sul, Brasil

DIMENSÃO 1	INDICADOR
Características sociodemográficas do autor da ação judicial - características da população em relação aos aspectos sociais e demográficos	1 - Proporção da população por faixa etária 2 - Proporção da população por ocupação 3 - Proporção da população por município de domicílio do autor da ação 4 - Renda familiar mensal
**DIMENSÃO 2**	**INDICADOR**
Características processuais das ações judiciais - aspectos que se encontram em conformidade com as leis nacionais e locais	1 - Tempo mediano de decisão liminar ou antecipação de tutela na primeira instância 2 - Tempo mediano de entrega do medicamento 3 - Proporção de concessão da liminar ou antecipação de tutela 4 - Proporção de sentenças favoráveis ao autor 5- Razão das ações judiciais coletivas 6 - Proporção de ações judiciais impetradas por tipo de réu da ação
**DIMENSÃO 3**	**INDICADOR**
Características médico-sanitárias das ações judiciais - aspectos relativos ao corpo de conhecimentos das Ciências da Saúde	1 - Proporção de medicamentos por subgrupos terapêutico/farmacológico/substância química 2 - Proporção de medicamentos prescritos pelo nome genérico 3 - Proporção de medicamentos requeridos que figuram nas listas de medicamentos essenciais vigentes 4 - Proporção de ações judiciais contendo documentos adicionais, que não a prescrição de medicamentos 5 - Proporção de medicamentos com força de recomendação Classes I e IIa na indicação terapêutica 6 - Proporção de diagnósticos principais, por categoria diagnóstica 7 - Proporção de pacientes com cadastro na instância de saúde, anterior a demanda judicial 8 - Razão de gasto de medicamentos demandados 9 - Proporção de medicamentos demandados com alternativa terapêutica no SUS
**DIMENSÃO 4**	**INDICADOR**
Características político-administrativas das ações judiciais - aspectos relacionados às competências executivas, administrativas e econômicas da Administração Pública. Neste caso refere-se à gestão da Assistência Farmacêutica no Sistema Único de Saúde	1 - Proporção de medicamentos registrados na ANVISA 2 - Proporção de medicamentos, por componente do bloco de financiamento da Assistência Farmacêutica 3 - Proporção de ações judiciais que possui ao menos um medicamento prescrito para indicação de uso “*off label*” 4 - Proporção de ações judiciais que demandam ao menos um medicamento que esteja fora dos componentes do bloco de financiamento da assistência farmacêutica 5 - Proporção de ações judiciais que demandam ao menos um medicamento do Componente Especializado da Assistência Farmacêutica

### Protocolo do estudo

Este protocolo foi disponibilizado na plataforma gratuita OSF (https://osf.io/32vyg). Os indicadores foram sintetizados e descritos segundo valores absolutos, proporções e razões. De acordo com a informação fornecida, eles foram classificados em quatro dimensões, relacionadas às características: 1) sociodemográficas do autor da ação; 2) político-administrativa; 3) processuais das ações judiciais; e 4) médico-sanitárias das ações.

### Análise dos resultados e estatística

Os dados foram coletados a partir das informações disponíveis no endereço https://esaj.tjms.jus.br/ e nos arquivos da DDIJ, referentes aos processos que atenderam aos critérios de inclusão.

Na dimensão 1, que analisa as características sociodemográficas do autor da ação, foram coletados dados como faixa etária em anos, ocupação, município de domicílio do autor da ação e renda familiar em salários mínimos. Para o cálculo de salários mínimos, foi considerado o valor vigente em cada ano analisado. As proporções foram calculadas pela razão entre o número de pacientes e a população total, multiplicada por 100.

Na dimensão 2, referente às características processuais das ações judiciais, foram apresentados os dados relativos ao tempo mediano de decisão liminar ou antecipação de tutela na primeira instância e para a entrega do medicamento, em dias. Calculou-se o valor mediano ou a média aritmética simples entre os dois valores médios da distribuição em ordem crescente.

As proporções de concessão da liminar ou antecipação de tutela, de sentenças favoráveis ao autor e de ações judiciais impetradas por tipo de réu da ação foram calculadas pela razão entre o número de ações relacionadas ao indicador e o número total de ações judiciais, multiplicada por 100.

Na dimensão 3, relacionada às características médico-sanitárias, os medicamentos foram classificados segundo a recomendação da Organização Mundial de Saúde, utilizando o Sistema Anatômico e Terapêutico Químico (código ATC)^(13)^ para a especificação quanto aos subgrupos terapêutico/farmacológico/substância química. A proporção foi calculada pela razão do número de medicamentos do subgrupo pelo número total de medicamentos demandados, multiplicada por 100.

Para o indicador “proporção de medicamentos requeridos que figuram nas listas de medicamentos essenciais vigentes”, foram utilizadas a RENAME 2020^(14)^, a Relação Estadual de Medicamentos Essenciais (RESME) 2020^(15)^ e a Relação Municipal de Medicamentos Essenciais (REMUME) 2016^(16)^. Para a proporção de diagnósticos principais, por categoria diagnóstica, a classificação foi feita de acordo com a décima revisão do Código Internacional de Doenças (CID-10).

Os medicamentos prescritos pelo nome genérico, as ações judiciais contendo documentos adicionais além da prescrição de medicamentos, e o número de pacientes com cadastro na instância de saúde, anterior à demanda judicial, foram verificados nos autos judiciais e expressos em percentuais.

A proporção de medicamentos com força de recomendação Classes I e II na indicação terapêutica e a com alternativa terapêutica no SUS, referentes aos medicamentos que tecnicamente poderiam ser intercambiáveis por outros medicamentos padronizados devido à equivalência terapêutica entre eles, foram expressas em percentuais. A razão de gasto com medicamentos demandados foi calculada com base nos empenhos emitidos para aquisição.

Para os indicadores da dimensão 4, que analisa as características político-administrativas das ações judiciais, foram avaliados os medicamentos registrados na Agência Nacional de Vigilância Sanitária (ANVISA) e com indicação de uso *off label*. Na análise dos medicamentos, por componente do bloco de financiamento da Assistência Farmacêutica, foi utilizada a RENAME 2020^(14)^, para verificar os itens não pertencentes e pertencentes ao Componente Especializado. Os cálculos relacionados a esta dimensão foram expressos em percentual.

## RESULTADOS

A Divisão de Dispensação de Insumos Judicializados (DDIJ) recebeu 676 processos com a determinação judicial para o fornecimento de medicamentos no período de julho de 2018 a junho de 2020. Ao analisar os indicadores propostos no Manual de Indicadores de Avaliação e Monitoramento das Demandas Judiciais de Medicamentos, observou-se que 87 (12,87%) processos continham todos os dados referentes aos indicadores elencados para análise, enquanto 589 (87,13%) apresentavam dados incompletos.

Os processos em geral continham uma ou mais solicitações de medicamento. Dessa forma, todos os 676 processos foram analisados, resultando em 1006 solicitações de medicamentos, com uma média de 1,49 medicamentos solicitados por processo. As solicitações envolviam 284 medicamentos com princípios ativos diferentes.

### Dimensão 1 - características sociodemográficas do autor da ação judicial

A maioria dos autores da ação judicial tinha idade entre 70 e 79 anos (14,20%), com uma média de idade de 48,85 anos, variando de três meses a 94 anos. Quase metade era aposentada ou pensionista (43,46%) e 10,87% eram do lar. A Tabela 1 totaliza 86,89% das ocupações dos autores da ação, incluindo apenas as ocupações com porcentagem maior ou igual a 5,00%.

**Tabela 1:** Características sociodemográficas do autor da ação judicial (N=676)

	n	%
Faixa etária (anos)		
Menor que 1 ano	4	0,59
1 a 9 anos	42	6,21
10 a 19 anos	43	6,36
20 a 59 anos	295	43,63
60 a 79 anos	186	27,51
80 anos ou mais	49	7,25
Sem informação	57	8,43
Ocupação		
Aposentado ou pensionista	236	43,46
Do lar	59	10,87
Desempregado	41	7,55
Estudante	29	5,34
Sem informação	133	19,67

Dos autores, 93,05% residiam em Campo Grande. Foi observada a ausência de informações quanto à faixa etária, ocupação e domicílio dos autores da ação em 57 (8,43%), 133 (19,67%) e 47 (6,95%) processos, respectivamente.

A renda familiar mensal média dos autores da ação foi de 1,47 ± 0,94 salários mínimos, com uma variação de 0,17 a 9,77 salários mínimos, sendo considerado o valor do salário mínimo de cada ano analisado. A falta de informação sobre a renda foi observada em 266 (39,35%) processos, conforme apresentado na Tabela 2. Não foi observada diferença significativa ao comparar as médias da renda entre os semestres analisados.

**Tabela 2:** Distribuição da Renda Familiar Mensal dos Autores das Ações Judiciais por Ano em Campo Grande, Mato Grosso do Sul, Brasil (2018-2020)

Renda	Ano 2018	Ano 2019	Ano 2020	Total	
	n	n	n	n	%
Até 0,5 salário mínimo	1	3	0	4	0,59
>0,5 a 1 salário mínimo	19	37	16	72	10,65
>1 a 3 salários mínimos	112	146	43	301	44,53
>3 a 5 salários mínimos	11	12	6	29	4,29
>5 a 11 salários mínimos	1	1	2	4	0,59
Sem informação	101	127	38	266	39,35
Total	245	326	105	676	100

*Salário Mínimo ano 2018 R$ 954,00^(17)^; Salário Mínimo ano 2019 R$ 998,00^(18)^; Salário Mínimo ano 2020 R$ 1.039,00^(19)^.*

### Dimensão 2 - características processuais das ações judiciais

Todas as ações tinham um único autor e tinham como réu a Secretaria Municipal de Saúde de Campo Grande-MS, não sendo observadas ações coletivas ou litisconsórcio no polo ativo durante o período estudado. A antecipação de tutela foi concedida em 600 processos (88,76%) e em 76 (11,24%) não houve registro desta ocorrência. O juiz concedeu liminarmente - logo no início da ação - o fornecimento dos medicamentos pleiteados em 1.005 (99,90%) solicitações. Em um processo, foi negada a concessão de acesso a um medicamento dentre os solicitados.

O tempo mediano entre o início do processo e a decisão que garantiu o acesso foi de 34 dias para os 616 processos que continham a informação. Nos 60 processos restantes, não havia informação quanto à data de início da ação judicial. Não houve diferença significativa ao comparar as médias de tempo entre o pedido e a decisão entre os semestres.

A entrega dos medicamentos postulados ocorreu em 419 (41,65%) solicitações, com tempo mediano entre a decisão e a entrega efetiva de 146 dias. As justificativas para a não entrega das 587 (58,35%) solicitações restantes foram: i) problemas de aquisição do medicamento em 380 solicitações (37,77%), ii) suspensão do tratamento pelo médico em 86 (8,55%), iii) óbito em 71 (7,06%) e iv) falta de contato com o paciente em 50 (4,97%).

### Dimensão 3 - características médico-sanitárias das ações judiciais

Nas 1006 solicitações, foram requisitados 284 medicamentos com princípios ativos diferentes. Desses, 255 foram classificados até o quinto nível da Classificação ATC. Houve predomínio de medicamentos que atuam no Sistema Nervoso (22,54%), Agentes Antineoplásicos e Imunomoduladores (16,20%), Sistema Digestório e Metabolismo (14,08%) e Sistema Cardiovascular (12,68%). Não possuíam classificação 20 medicamentos (7,04%), dos quais três (1,05%) foram classificados até o quarto nível e cinco (1,76%) até o terceiro.

Em 547 (54,37%) das 1006 solicitações, os medicamentos estavam prescritos pelo nome genérico, 911 (90,56%) possuíam indicação terapêutica para o diagnóstico mencionado nos autos, e 944 (93,80%) dispunham de alternativa para o tratamento no SUS.

Adicionalmente, em 567 (83,88%) das 676 ações, foi possível identificar documentos adicionais, como exames específicos e laudos médicos, para o embasamento do diagnóstico e da solicitação. Não foram identificadas informações referentes ao cadastro dos pacientes em instância de saúde anterior ao processo judicial. Os pareceres do Núcleo de Apoio Técnico (NAT Jus) estavam presentes em 868 (86,28%) das solicitações, sendo a maioria desfavorável à concessão do medicamento, 532 (52,88%), e 336 (33,40%) pareceres favoráveis. O parecer não estava presente em 138 (13,72%) das solicitações de medicamentos. A proporção de processos de pacientes oriundos de tratamento no SUS foi de 385 (56,95%), 220 (32,54%) de outros serviços e 71 (10,50%) sem este dado.

A Tabela 3 apresenta os dados sobre a presença nas listas oficiais citadas para os 284 medicamentos solicitados nos processos. Noventa e nove (34,86%) medicamentos estão presentes na RENAME, sendo que hidralazina, lactulose, eculizumabe e acetato de hidrocortisona estão presentes apenas nesta lista. Bromoprida, diosmina com hesperidina, cilostazol e fenoterol são contemplados somente na REMUME. A dapagliflozina e o sacubitril com valsartana estão presentes apenas na RESME. Há medicamentos que pertencem concomitantemente a todas as listas, como as insulinas glargina e asparte e o ciprofibrato.

**Tabela 3:** Inclusão dos Medicamentos Requisitados Judicialmente nas Listas Oficiais de Medicamentos em Campo Grande, Mato Grosso do Sul, Brasil (2018-2020)

Listas oficiais	n	%
RENAME	99	34,86
REMUME	57	20,07
RESME	41	14,43
RENAME, REMUME E RESME	3	1,06
Não pertence	183	64,44
Total	284	100

*RENAME - Relação Nacional de Medicamentos Essenciais; REMUME - Relação Municipal de Medicamentos Essenciais; RESME - Relação Estadual de Medicamentos Essenciais.*

O somatório das três listas oficiais de padronização de medicamentos não totaliza 100% porque há medicamentos que pertencem a apenas uma lista.

Os medicamentos mais solicitados foram a enoxaparina 40mg (86; 8,55%), brometo de tiotrópio 2,5mcg (69; 6,86%), ribaroxabana 20mg (64; 6,36%) e as insulinas glargina e glulisina (74; 7,35%).

Em 626 (92,60%) processos foi possível identificar pelo menos um diagnóstico, perfazendo um total de 244 morbidades. Havia processos com menção a vários CID. A falta desta informação foi observada em 50 (8,40%) processos. As morbidades mais frequentes estão dispostas na Tabela 4.

**Tabela 4:** Distribuição dos Principais Diagnósticos nos Processos Judiciais Segundo o Código Internacional de Doenças (CID-10) em Campo Grande, Mato Grosso do Sul, Brasil (2018-2020)

CID	Segundo semestre 2018	Primeiro semestre 2019	Segundo semestre 2019	Primeiro semestre 2020	Total	%
C61 - Neoplasia Maligna da Próstata	8	2	4	3	17	1,69
D68 - Defeitos da Coagulação	23	26	14	9	72	7,16
E10 - Diabetes Mellitus Insulinodependente	28	19	22	11	80	7,95
F33 - Transtorno Depressivo Recorrente	7	1	2	5	15	1,49
I10 - Hipertensão Essencial (Primária)	13	7	5	6	31	3,08
J44 - Outras Doenças Pulmonares Obstrutivas Crônicas	19	8	34	5	66	6,56
J45 - Asma	7	5	1	3	16	1,59
M79 - Outros Transtornos dos Tecidos Moles, Não Classificados em Outra Parte	10	-	2	3	15	1,49

O custo médio dos medicamentos fornecidos para as 419 solicitações, de acordo com os empenhos emitidos, foi de R$ 399,45. O ocrelizumabe e o alemtuzumabe foram os medicamentos com a dose mais cara, R$ 25.285,98 e R$ 24.882,05, respectivamente. A gliclazida possui o comprimido mais barato, custando R$ 0,04. Considerando o tratamento mensal, o custo médio foi de R$ 2.183,68.

O repasse de recursos financeiros federais é de R$ 5,90 por habitante/ano, além das contrapartidas estadual e municipal, que devem ser de no mínimo R$ 2,36 por habitante/ano (14). Assim, o investimento anual para aquisição de medicamentos por cidadão campo-grandense é de, no mínimo, R$ 10,62. O custo para a concessão do tratamento mensal por via judicial foi 2.467 vezes maior que este valor mínimo.

### Dimensão 4 - características político-administrativas das ações judiciais

A distribuição dos medicamentos segundo o componente do bloco de financiamento da Assistência Farmacêutica é detalhada na Tabela 5. Nota-se um predomínio (64,79%) de solicitações de medicamentos que não estão incluídos nos componentes. De acordo com a RENAME, 100 medicamentos (35,21%) fazem parte dos componentes do bloco de financiamento, e alguns medicamentos figuram em mais de um componente simultaneamente.

**Tabela 5:** Classificação dos Medicamentos Requisitados Judicialmente por Bloco de Financiamento do SUS em Campo Grande, Mato Grosso do Sul, Brasil (2018-2020)

Medicamento por bloco de financiamento	n	%
Básico	52	18,31
Estratégico	6	2,11
Especializado	41	14,44
Básico e Especializado	1	0,35
Fora dos componentes de financiamento	184	64,79
Total	284	100

As ações judiciais que demandaram ao menos um medicamento não incluído nos componentes do bloco de financiamento somaram 443 (65,53%), e as que solicitaram pelo menos um medicamento do Componente Especializado totalizaram 245 (36,24%).

Todos os 284 medicamentos possuem registro na ANVISA, e três deles tinham indicação *off label*. O rituximabe, o pembrolizumabe e o canabidiol não têm indicação na bula para lúpus eritematoso sistêmico, pênfigo foliáceo, pênfigo vulgar grave, neoplasia maligna do olho e anexos e doença de Parkinson, conforme os diagnósticos mencionados nos autos.

## DISCUSSÃO

Apesar de o Manual de Indicadores de Avaliação e Monitoramento das Demandas Judiciais de Medicamentos colaborar com gestores da saúde e do sistema judicial na identificação de dificuldades, no desenvolvimento de estratégias e no aprimoramento de instrumentos e/ou mecanismos para melhorar a assistência farmacêutica e reduzir o volume de ações judiciais, são escassas as publicações locais que utilizam esses indicadores, assim como avaliações nacionais sobre a qualidade da ação e da decisão judicial.

Além disso, a indisponibilidade dos dados, tanto completa quanto parcial, foi constatada em 87,13% dos processos. A ausência de rastreabilidade após a decisão judicial e a falta de análise dos dados presentes nos processos prejudicam o dimensionamento do impacto, dos custos, do investimento, da possibilidade de padronização de novos medicamentos e o aprimoramento da assistência farmacêutica. A rastreabilidade após a decisão judicial incluiria informações sobre o recebimento do medicamento, o uso pelo paciente e a eficácia do tratamento. Quanto à análise dos dados, é importante informar que foi realizada nesta pesquisa como pré-requisito para obtenção de grau em pós-graduação stricto sensu (mestrado), mas que, em condições rotineiras, tal análise não é uma prática do serviço judiciário. Assim, a inexistência de rotinas de coleta, processamento e análise de dados dificulta as tomadas de decisão pelos entes públicos para reduzir o impacto da judicialização em seus orçamentos^(9)^.

Na análise das características processuais das ações judiciais, houve a concessão para a maioria dos medicamentos requeridos. Apenas uma solicitação foi negada, pois havia alternativa padronizada na RENAME e não havia comprovação, por meio de laudo médico, da imprescindibilidade do medicamento prescrito ou da ineficácia do fármaco fornecido pelo SUS. Essa decisão contrária ao fornecimento do medicamento prescrito, baseada na análise multidisciplinar conforme consta no parecer dos autos, e na verificação junto às listas oficiais da presença de alternativa padronizada no SUS, possibilita ações efetivas além de promover a otimização dos recursos aplicados^(20-21)^.

A morosidade e o baixo percentual de fornecimento dos medicamentos comprometem os tratamentos, principalmente daqueles pacientes que se encontram em estado avançado da doença e sob risco de óbito. As exigências e os prazos inerentes ao processo de aquisição dos medicamentos podem justificar essa demora no cumprimento da demanda. Portanto, o conhecimento desta burocracia, bem como do tempo para o fornecimento do medicamento, deve ser considerado pelo judiciário na emissão do veredicto, assim como pelo médico e pelo paciente para evitar o comprometimento do tratamento.

Nos resultados da dimensão três, a prescrição pelo nome genérico do medicamento, proveniente do SUS, foi observada na maioria das ações, assim como em ações no Rio de Janeiro^(22)^ e no Distrito Federal^(23)^. O contrário foi evidenciado nas ações dos estados do Rio Grande do Norte^(24)^ e de Minas Gerais^(5)^, onde as prescrições originavam-se, principalmente, dos serviços particulares de saúde. Esse predomínio de prescrições oriundas do SUS sugere o desconhecimento ou a não adesão dos profissionais prescritores às listas oficiais e aos protocolos clínicos. A falta de educação continuada, com a divulgação e o suporte sobre as diretrizes clínicas e os medicamentos padronizados, pode induzir ao aumento da judicialização. O acesso irregular aos medicamentos disponibilizados pelo SUS é outro fator agravante.

Na maioria dos processos judiciais, houve a presença de documentos adicionais. Esses documentos podem direcionar a atualização e a incorporação de novos medicamentos às listas oficiais e a implementação de protocolos clínicos, uma vez que podem conter informações sobre a ineficácia do fármaco fornecido pelo SUS e a nova conduta médica proposta.

A presença de pareceres do NAT Jus, majoritariamente desfavoráveis devido à existência de alternativa padronizada no SUS ou à falta de comprovação da imprescindibilidade do medicamento prescrito, mostra que as recomendações nem sempre são acatadas, uma vez que houve concessão da maioria dos medicamentos. Contudo, fornecer o tratamento solicitado não garante a efetividade pretendida e pode intensificar a judicialização. Decisões judiciais informadas por evidências e a utilização de protocolos clínicos como parâmetro técnico contribuem para o fornecimento de medicamentos seguros e eficazes para a população^(6,25)^.

Grande parte dos medicamentos solicitados não está presente nas listas oficiais citadas. O mesmo foi constatado por estudos nos estados de São Paulo^(4)^, Minas Gerais^(5)^ e no Rio Grande do Norte^(24)^. No entanto, há medicamentos presentes apenas na RENAME que, por integrarem a relação nacional, têm recursos financeiros destinados à sua aquisição e poderiam ser incorporados à lista de medicamentos local.

A adoção de relações específicas e complementares de medicamentos é legalmente amparada^(26)^ e deve atender aos principais problemas de saúde e programas locais. A inclusão de medicamentos exclusivamente na REMUME e na RESME, como observado nesta pesquisa, otimiza os recursos investidos na aquisição de medicamentos e amplia o acesso, já que as compras poderão atender a um número maior de pacientes, aumentando a quantidade adquirida e, consequentemente, a concorrência nos processos licitatórios.

As falhas na logística de aquisição de medicamentos, o desconhecimento dos prescritores das listas oficiais e das exigências para obtenção do tratamento também são justificativas para o requerimento por via judicial. Isso pode ser demonstrado com os medicamentos mais solicitados como a enoxaparina e as insulinas glargina e glulisina que estão presentes na RESME e possuem exigências para sua obtenção presentes nos Protocolos Clínicos e Diretrizes Terapêuticas (PCDT).

Condições crônicas como diabetes mellitus, doenças pulmonares obstrutivas crônicas e doenças hipertensivas foram os diagnósticos mais frequentes, como também foi evidenciado em São Paulo^(4)^ e no Rio Grande do Norte^(24)^. O predomínio de pacientes idosos encontrado na pesquisa, juntamente com o envelhecimento da população em geral, pode justificar esses diagnósticos frequentes, a necessidade de tratamentos contínuos^(4)^ - na maioria das vezes de alto custo - e os requerimentos por via judicial.

Além disso, os resultados indicam a judicialização para acesso a medicamentos relacionados a tratamentos de condições crônicas que requerem cuidados primários e que poderiam ter sido resolvidos na Atenção Primária.

De acordo com os empenhos emitidos para a aquisição dos medicamentos, o custo para concessão do tratamento mensal por via judicial foi 2.467 vezes maior que o investimento para aquisição de medicamentos. Essa alocação de recursos se contrapõe ao princípio da equidade, podendo ocasionar desigualdades de acesso, uma vez que prioriza necessidades individuais e desconsidera a coletividade. A política de Assistência Farmacêutica parece não ser observada, o que pode prejudicar o planejamento e administração dos recursos, que são escassos e devem ser delineados pelas políticas de saúde.

Nas características político-administrativas das ações judiciais analisadas, observou-se o uso *off label* de três medicamentos, considerando os diagnósticos mencionados nos autos e a bula. O uso *off label* de um medicamento pode implicar que ele não produza o efeito esperado, seja ineficaz ou inseguro. A concessão para este fim pode requerer uma avaliação multiprofissional abrangente para verificar se o pedido é justificável tecnicamente e terapeuticamente, além do monitoramento de farmacovigilância para identificar, avaliar e monitorar a ocorrência de eventos adversos e garantir que os benefícios sejam maiores que os riscos^(27)^.

Assegurar o adequado acompanhamento do paciente, juntamente com a concessão do fornecimento do medicamento por via judicial, colabora com a garantia do efetivo acesso à saúde e à justiça, promovendo o uso racional dos medicamentos e evitando possíveis fraudes ou má aplicação de recursos públicos. Monitorar os desfechos das demandas de saúde, obter dados relativos à necessidade ou não da manutenção do benefício e mediar a relação entre usuário demandante e sistema judiciário pode levar a uma diminuição em torno de 30% dos custos globais^(28-29)^.

Mesmo contemplando necessidades individuais, a judicialização ainda pode gerar efeitos positivos, dado que induz a discussão sobre a atualização dos programas e protocolos. Isso possibilita a oportunidade de análises para a incorporação de novos medicamentos às listas oficiais ou reparação das falhas relacionadas à aquisição de medicamentos.

Apesar de existirem estratégias para a qualificação da administração pública brasileira para abordar a judicialização da assistência à saúde, estas não são obrigatórias e não têm garantias de implementação por parte das instituições públicas^(30)^. Reforçamos a possibilidade de uso dos Indicadores de Avaliação e Monitoramento das Demandas Judiciais de Medicamentos como ferramenta de aproximação entre gestores e o poder judiciário para análise dos processos e aprimoramento das políticas relacionadas à saúde, que promovam o acesso integral aos cuidados de saúde e melhorem a distribuição de bens sociais e serviços públicos entre a sociedade^(11-12,31)^.

Mesmo existindo uma lacuna de conhecimentos técnicos específicos dos juízes, a solicitação de informações baseadas em evidências e a utilização de protocolos como parâmetro podem proporcionar maior segurança aos julgadores, pois haverá um referencial técnico objetivo explícito, capaz de impactar na reformulação da própria política pública de saúde^(32)^.

A ampla divulgação dos dados das avaliações das demandas judiciais, das listas oficiais de medicamentos, a elaboração e a implementação de cartilhas e manuais para o direcionamento da prática clínica poderiam otimizar a alocação de recursos, contribuir para a ampliação do acesso à saúde e possibilitar a redução da intensidade da judicialização.

A integração das equipes multiprofissionais de saúde e jurídicas, com a solicitação de informações baseadas em evidências, além da comunicação sobre a continuidade ou suspensão do tratamento requerido através da demanda judicial, traz efetividade ao acesso tanto jurídico quanto de saúde.

### Limitações do estudo

A falta de acesso à prescrição médica ou à sua cópia e a outros documentos médicos apensados ao processo judicial foram importantes limitações desta pesquisa. Vale ressaltar que a Secretaria Municipal de Saúde não possui sistematização dos processos judiciais, e a construção do banco de dados foi realizada manualmente através de cópias digitalizadas dos processos judiciais, o que pode acentuar as lacunas evidenciadas na pesquisa.

### Contribuições para a área da enfermagem, saúde e políticas públicas

O estudo contribui significativamente para a área da enfermagem ao evidenciar que, com o conhecimento das peculiaridades das demandas judiciais, é possível estruturar melhor o cumprimento destas. O fornecimento do medicamento, objetivo principal da demanda, pode ser acompanhado por um suporte contínuo ao usuário por toda a equipe de saúde, promovendo o uso racional e seguro do medicamento. A implementação de protocolos práticos e a capacitação dos profissionais prescritores, sensibilizando-os quanto aos tratamentos disponíveis no SUS, também contribuem para a organização dos serviços de saúde e das políticas relacionadas ao medicamento.

Já para a área de saúde e para as políticas públicas, a avaliação dos medicamentos e dos protocolos para atualizações frequentes, bem como a sistematização de todo o processo de trabalho, ao criar rotinas de análise de dados das demandas judiciais e promover diálogos entre gestores e o poder judiciário, contribuem para a otimização dos recursos empregados.

## CONCLUSÕES

A análise das demandas judiciais no município de Campo Grande, através dos indicadores elencados, traz subsídios para comparações com outras localidades e para o dimensionamento da judicialização no município. Apesar das limitações do estudo, com o conhecimento das peculiaridades das demandas, há colaboração para o direcionamento das políticas relacionadas aos medicamentos e a redução da judicialização.

Entretanto, novas pesquisas avaliativas ou de *benchmark* que avaliem os indicadores relacionados às demandas judiciais para acesso a medicamentos são úteis para o aprimoramento do acesso a medicamentos e a otimização dos recursos empregados para seu cumprimento, levando as equipes de saúde a promover o uso racional e seguro dos medicamentos fornecidos.
